# Generative AI, cyberchondria, and digital health inequality: a public health challenge for Somalia and other fragile health systems

**DOI:** 10.3389/fpubh.2026.1942140

**Published:** 2026-10-27

**Authors:** Abdisamad Abdihakim Aadam, Maida Ali Daahiye, Abdirizak Ahmed Mohamed, Abdimalik Dahir Ali, Ilyas Abdullahi Khalif

**Affiliations:** 1 Faculty of Health Sciences and Tropical Medicine, Somali National University, Mogadishu, Somalia; 2 Benaadir Research, Consultancy and Evaluation Center, Mogadishu, Somalia; 3 Center for Health Research and Innovation, Somali National University, Mogadishu, Somalia; 4 Faculty of Health Sciences, Salaam University, Mogadishu, Somalia

**Keywords:** cyberchondria, digital health, fragile health systems, generative artificial intelligence, health inequality, health misinformation, large language models, Somalia

## Abstract

Generative artificial intelligence (GenAI) is transforming access to health information through rapid, personalized, and conversational responses. While this may improve health education and reduce information barriers, it may also increase exposure to misinformation, uncertainty, misplaced trust, and health anxiety. This Perspective examines the potential relationship between GenAI-mediated health-information seeking, cyberchondria, digital health inequality, and health-system vulnerability, using Somalia as an important context for examining how these dynamics may unfold in a fragile health system. We argue that conversational AI may reinforce existing patterns of repeated symptom searching and reassurance seeking, although direct evidence linking GenAI to cyberchondria remains limited. Somalia-specific evidence on GenAI-mediated health-information seeking is currently unavailable; therefore, the Somali context is considered through structural conditions, including unequal digital access, constrained healthcare access, and limited local health-information infrastructure. Safe and equitable adoption requires Somali-language validation, digital-health literacy, human oversight, data protection, and local research capacity.

## Introduction and context

Digital health has transformed how people encounter, interpret, and act on health information. The internet now functions as a primary health-information source for many users, and health-related searching is often experienced as both accessible and empowering, particularly when formal care is difficult to reach (1, 2). Yet this expansion has also created new risks. Repeated online symptom searching can escalate medical concerns and distress rather than resolve them (1–3). Cyberchondria refers to excessive or repetitive online health-information seeking that can intensify health anxiety, uncertainty, and reassurance-seeking, particularly when individuals repeatedly search for information about perceived symptoms or illnesses (1, 2).

The emergence of generative artificial intelligence introduces a qualitatively different health-information environment. Unlike conventional search engines or static websites, conversational AI systems do not simply retrieve existing sources; they synthesize probabilistic information into fluent, tailored responses that can appear direct, coherent, and authoritative to users seeking explanations for symptoms or guidance about next steps (4, 5). This matters for public health because generative AI is rapidly expanding into routine consumer use, while evidence also shows that such systems can produce errors and spread misleading medical content at scale, and that patients may act on those responses in ways that affect individual care and broader health systems (4, 5). Beyond misinformation alone, generative AI may alter how people search for and interpret health information by providing rapid, conversational, and potentially inaccurate responses, raising concerns about an emerging AI-driven infodemic and its implications for health-information seeking (6). In this setting, the long-recognized drivers of cyberchondria—information overload, uncertainty, unrealistic expectations of diagnostic closure, and confusion about source trustworthiness—remain relevant, but generative AI may plausibly interact with these processes by delivering fast, personalized, and seemingly definitive answers rather than ranked links (1, 7).

Digital health technologies may not benefit all population groups equally, particularly where access to devices, connectivity, digital skills, and health resources is uneven (8, 9). Fragile and conflict-affected settings face additional constraints, including weak technological infrastructure, limited computer literacy, privacy concerns, and underdeveloped health information systems, even as digital health technologies offer promising routes to expand access to health information and care (10). These concerns are sharpened when AI systems are developed in high-income contexts and deployed elsewhere with embedded assumptions that may not travel well, especially where representative data and locally grounded health-information resources are limited (9, 11).

Somalia provides an important context for considering how generative AI may interact with existing inequalities in access to health information, misinformation, uncertainty, and health-system capacity within a constrained health-information ecosystem. This Perspective examines the potential interaction between generative AI, cyberchondria, digital health inequality, and health-system vulnerability, using Somalia to illustrate structural conditions that may shape these dynamics and to identify questions relevant to other fragile and resource-constrained settings.

## Literature selection

This Perspective was informed by a focused narrative literature search conducted primarily through Google Scholar. Searches combined terms related to “generative AI,” “large language models,” “health information seeking,” “cyberchondria,” “digital health inequality,” “health misinformation,” “Somalia,” “fragile health systems,” and “LMICs.” Additional targeted searches addressed digital connectivity, healthcare access, and socioeconomic disparities in Somalia and the wider Horn of Africa. Reference lists of relevant publications were also screened.

Studies were considered relevant if they addressed GenAI and health-information seeking, cyberchondria, digital health inequality, health-system constraints in fragile or low-resource settings, or Somalia-specific digital-health evidence. Priority was given to peer-reviewed studies, systematic or scoping reviews, methodological studies, and authoritative reports. Publications unrelated to these domains or lacking substantive evidence were excluded.

Because this was a Perspective rather than a systematic review, screening focused on conceptual and empirical relevance, without formal risk-of-bias assessment or quantitative synthesis. The search was not intended to be exhaustive, and publication or selection bias cannot be excluded. No nationally representative estimate of GenAI-mediated health-information seeking in Somalia was identified; therefore, the Somalia-specific discussion addresses structural conditions that may shape emerging use rather than measured population-level behavior. The proposed relationships in Figure 1 should consequently be interpreted as hypothesis-generating rather than established causal relationships.

**Figure 1:**
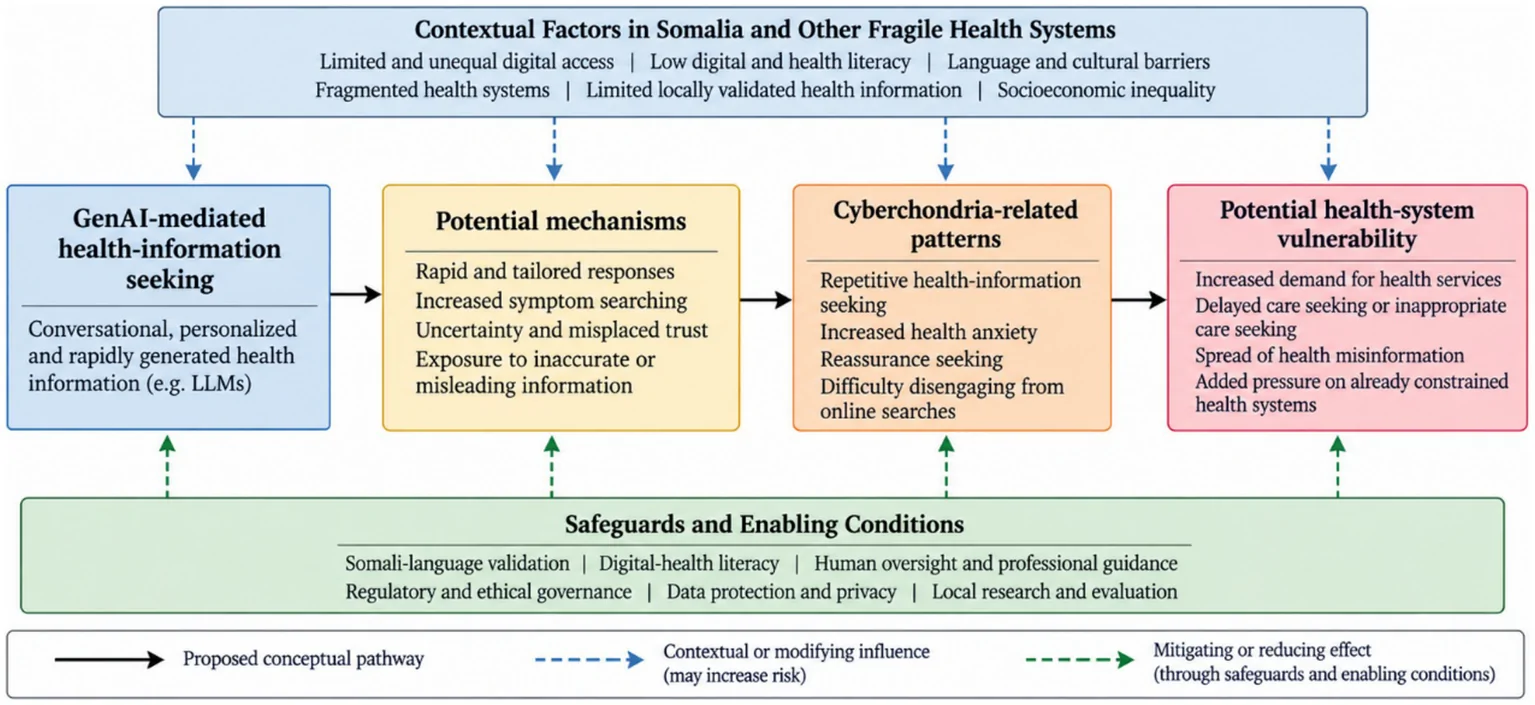
Proposed conceptual framework linking generative AI-mediated health-information seeking, cyberchondria-related patterns, digital health inequality, and potential health-system vulnerability in Somalia and other fragile health systems. The framework is hypothesis-generating; arrows represent proposed conceptual relationships rather than established causal effects.

## Generative AI and cyberchondria

Generative AI may modify, rather than simply extend, the relationship between online health-information seeking and cyberchondria, although this proposed relationship has not yet been established empirically (1, 12). Conversational systems introduce a different informational setting because they provide rapid, fluent, and personalized responses, support iterative questioning, and are often used for clarity and convenience even when users remain uncertain about accuracy or sourcing (13, 14).

This interaction style could lower the threshold for repeated symptom exploration and may increase the persuasive force of the information returned, although these effects require empirical investigation (13, 15). At the same time, large language models can generate plausible but unsupported content, omit relevant guidance, produce variable answers across identical prompts, and display overconfidence despite limited reliability (16, 17).

Direct evidence that generative AI causes cyberchondria is not yet established. The more defensible hypothesis is mechanistic: when users seek symptom explanations from systems that are conversational, fast, and apparently authoritative but also probabilistic, incomplete, and sometimes contradictory, unresolved uncertainty could persist or potentially intensify rather than diminish. In individuals prone to health anxiety, this could plausibly contribute to the familiar cyberchondria pathway from symptom concern to repeated checking, transient reassurance, renewed doubt, and escalating distress (1).

## Contextual differences in fragile health systems

Fragile and resource-constrained health systems may experience different consequences from GenAI-mediated health-information seeking because digital access can expand faster than system capacity to interpret, validate, and respond to what people find online. Across LMICs, digital health adoption has accelerated, but scale-up is often limited by fragmented ecosystems, weak interoperability, uneven infrastructure, scarce skilled labor, and institutional unpreparedness rather than by lack of technological interest alone (18, 19). Where access to clinicians is uneven and referral pathways are weak, AI-generated information can carry more practical weight because opportunities for timely professional interpretation are reduced, while digital health systems themselves may lack the registries, data quality, and coordination needed to contextualize information across levels of care (10, 20).

The consequences also vary because digital and health literacy are unequally distributed. Limited connectivity, device access, language fit, and user proficiency can hinder participation, while low digital health literacy reduces people’s ability to judge source quality and can deepen health inequities as care and information environments become more digitized (21, 22). At the system level, weak regulatory clarity, incomplete governance, and dependence on informal or poorly integrated information channels can further widen the gap between information exposure and institutional capacity to assure quality, accountability, and follow-up (19, 22). These dynamics are heterogeneous across settings, but they are likely to be most consequential where technological exposure, constrained healthcare access, and thin health-information infrastructures intersect. Somalia therefore provides a relevant real-world context for examining how digital health inequality and health-system vulnerability may shape the potential emergence and consequences of GenAI-mediated health-information practices (10).

## Somalia as a context for digital health inequality

Somalia provides a relevant context for examining the structural conditions under which emerging digital and AI-mediated health-information practices may develop, given expanding digital connectivity, a fragmented health system, and limited capacity to validate and regulate new sources of health information. Mobile phones and mobile-based services are widely used, but internet access remains highly unequal. A recent national study reported internet access of 9.3% overall, compared with 19.8% in urban areas and 3.6% among rural and nomadic populations; access also ranged from approximately 1% among the poorest households to 22% among the wealthiest (23). Although mobile-money use was 85.2%, this does not imply equivalent access to internet-dependent digital health information or AI-enabled services (23). These conditions may shape the future diffusion of GenAI-mediated health information, but they do not constitute evidence of current GenAI use. No nationally representative estimate of GenAI-mediated health-information seeking in Somalia was identified in the literature reviewed for this Perspective. The Somalia-specific discussion therefore focuses on structural factors—including connectivity, socioeconomic inequality, healthcare access, digital literacy, and availability of locally validated health information—that may influence emerging use (23–25). Distance to health facilities was reported as a major problem by 64.0% of respondents overall and 70.0% of rural and nomadic populations (23).

This finding suggests that digital technologies may complement but cannot replace physical healthcare access. Whether GenAI currently affects health-information seeking, health anxiety, or cyberchondria among Somali populations remains an empirical question requiring further research (23).

## Potential benefits of generative AI in resource-constrained health systems

Generative AI holds significant potential utility for expanding access to health information, supporting health education, and reducing some informational barriers, particularly when appropriately adapted to local contexts (26, 27). In health education, AI tools may enhance health literacy by providing personalized, adaptive learning that simplifies complex medical concepts for diverse populations (26). For patient information, generative models can translate scientific findings into accessible formats, potentially improving patient comprehension and engagement (28, 29). For healthcare workers, generative AI may reduce administrative burdens and support clinical decision-making, potentially freeing time for direct patient care (30).

However, these benefits represent potential utility rather than demonstrated effectiveness, and evidence on real-world implementation remains limited and context-dependent (31, 32). Realizing these benefits depends on accuracy, clinical validation, appropriate localization, and language quality (4). Furthermore, effective deployment requires digital literacy, human oversight, privacy protection, and robust governance frameworks (30). Without these safeguards, the deployment of generative AI risks exacerbating existing inequities and introducing new pathways for misinformation, underscoring the need for careful examination of the risks that follow (32, 33).

## GenAI health information risks

Generative AI may widen access to health information while also creating new pathways of harm through factual error, misplaced trust, and unequal capacity to evaluate outputs. Established evidence shows that large language models can generate false or misleading content, including in medical contexts, and that fluency does not reliably signal truth (15, 34). In oncology-related evaluations, a meta-analysis of 39 studies reported an overall hallucination incidence of approximately 23%, with variation across prompting contexts (35). Separately, multi-model clinical testing under deliberately adversarial conditions reported substantially higher hallucination rates of 50–82%, even when mitigation prompts reduced but did not eliminate errors (36).

These estimates are not directly comparable because they derive from different tasks, prompting conditions, evaluation designs, and exposure scenarios. Importantly, neither estimate should be interpreted as the prevalence of hallucinations during routine consumer use.

The lower estimate illustrates that hallucinations can occur under evaluated health-information conditions, whereas the higher estimates demonstrate that model vulnerability may increase under deliberately challenging conditions (35, 36).

These systems can also be exploited to generate credible-sounding misinformation at scale, and they may propagate false details embedded in prompts, making both inadvertent error and deliberate manipulation relevant to public health (36).

Established evidence from the pre-GenAI literature shows that repeated online health searching is associated with health anxiety, information overload, reassurance seeking, and disruption of usual care relationships (1). Studies further show that online information overload and information trust can trigger cyberchondria, and that cyberchondria is associated with self-medication, health-information fatigue, and lower therapy compliance (37, 38). It is therefore plausible, but not yet definitively proven, that AI-mediated symptom seeking could intensify unnecessary consultations or testing by producing authoritative but uncertain explanations that users seek to verify offline. That pathway remains a research hypothesis, but it is consistent with evidence that cyberchondria can alter patterns of healthcare use and that patient-generated digital demands already add to physician work outside working hours (1, 39).

Evidence from digital health and online health-information research is not uniformly negative. For some users, accessible health information may provide reassurance, improve clinical understanding, support informed decision-making, and reduce barriers to appropriate care (40, 41). These potential benefits mean that AI-mediated health-information seeking should not be equated with cyberchondria. Rather, the concern is that, in individuals with high health anxiety or repeated reassurance-seeking tendencies, the same accessibility and interactivity could plausibly contribute to escalating searches when uncertainty remains unresolved (1). Thus, the public-health implications of GenAI-mediated health information are likely to depend on user characteristics, the quality and framing of AI outputs, and the availability of reliable clinical alternatives (40, 41).

The equity implications are equally important. AI can reduce information inequality, but benefits depend on digital literacy, access to devices and connectivity, language fit, and the quality of the model deployed (42, 43).

Current evidence shows that inadequate representation in training data, poor transportability across populations, and deployment without local adaptation can reproduce or amplify disparities, especially when tools built in high-income settings encode assumptions that do not travel well (11, 33).

Language inequity is part of this problem: multilingual or low-resource populations may face worse performance, lower comprehensibility, or exclusion from validation altogether, while people with limited English proficiency and older or poorer users already experience reduced access across digital care environments (11, 42). Privacy risks also rise when users disclose sensitive symptoms or records to proprietary systems, particularly where data protection and audit capacity are weak (7, 11).

The central paradox is that GenAI may reduce information scarcity while simultaneously reproducing health inequality. Where health systems lack the capacity to validate outputs, absorb avoidable demand, protect data, and monitor unequal model performance, even modest individual-level errors can accumulate into system-level strain. That prospect makes governance, validation, and equity-centered oversight not optional complements to innovation, but prerequisites for safe public-health deployment (15, 42).

## Governance and policy roadmap

A realistic governance roadmap for Somalia and similar fragile health systems should prioritize safe access over premature restriction and build capacity in stages rather than assume mature regulatory institutions already exist. In the immediate term, the most feasible actions are low-cost public guidance on appropriate uses and limits of GenAI for health, basic AI and digital-health literacy for frontline workers and the public, and simple transparency norms requiring disclosure when health information is AI-generated, because weak skills, governance gaps, and user-centered design failures are recurring barriers in LMIC digital health deployment (32, 33).

Medium-term priorities should focus on local validation and institutionalization: small multidisciplinary oversight groups within ministries, universities, and professional bodies; Somali-language and medical-translation evaluation; minimum clinical safety standards for public-facing tools; and integration into national digital-health strategies rather than stand-alone pilots (32, 33). Digital health adoption should also be understood as a process of managing competing tensions rather than simply maximizing technological access. Recent work on telemedicine highlights tensions between access and quality, innovation and system stability, and user needs and system demands, suggesting that sustainable digital transformation requires attention to these competing dimensions (44). This is also the stage for practical misinformation monitoring and shared reporting channels, since effective infodemic response depends on regulatory coherence, interoperable public-health infrastructure, and interdisciplinary collaboration (45).

Long-term priorities require durable governance capacity: independent auditing, bias and fairness assessment across population groups, Somali medical-language datasets, stronger health data governance, and domestic research capacity linked to sustainable financing and accountability (11).

Public trust should also be treated as an important component of responsible AI-enabled health services. Recent research conceptualizes trust in AI-enabled telemedicine as multidimensional, involving affective, cognitive, and structural dimensions related to user experience, perceived competence, transparency, and institutional safeguards (46). In fragile health systems, strengthening trust may therefore require not only improving the technical performance of AI tools but also establishing clear accountability, transparent communication, and credible mechanisms for oversight and redress.

The policy logic is therefore cumulative: start with guidance, literacy, and human oversight; then build validation, coordination, and monitoring; and only then move toward more advanced forms of AI governance, independent evaluation, and scaled integration into health systems (32, 33).

Key quantitative indicators of digital-health inequality and pragmatic governance priorities for the safe and equitable use of generative AI in health-information contexts are summarized in Table 1.

**Table 1:** Quantitative indicators of digital-health inequality and pragmatic governance priorities for the safe and equitable use of generative AI in health-information contexts in Somalia.

Section	Indicator/priority area	Estimate/recommended action	Context/time horizon	Reference
A. Digital-health inequality indicators	Internet access	9.3%	Overall access	(23)
	Internet access – urban	19.8%	Higher urban access	(23)
	Internet access – rural/nomadic	3.6%	Substantially lower access	(23)
	Internet access – poorest households	1.0%	Socioeconomic gradient	(23)
	Internet access – wealthiest households	22.0%	Socioeconomic gradient	(23)
	Mobile-money use	85.2%	High mobile-financial-service penetration despite low internet access	(23)
	Distance to health facility as a major problem	64.0%	Persistent physical-access barrier	(23)
	Rural/nomadic reporting distance as a major problem	70.0%	Greater physical-access constraint	(23)
	LLM hallucination in oncology evaluations	≈23%	Evaluation-specific; not Somalia-specific prevalence	(35)
	Hallucination under adversarial clinical conditions	50–82%	Specific clinical decision-support testing conditions	(36)
B. Pragmatic governance priorities	AI and digital-health literacy	Develop simple Somali-language guidance on AI limitations, verification, and when to seek professional care	Short term	(32, 33)
	Somali-language safety and validation	Develop and test Somali medical prompts, translations, and safety benchmarks	Short–medium term	(11, 24, 25)
	Human oversight and clinical safeguards	Distinguish AI-generated information from diagnosis and clinical decision-making; encourage professional referral for high-risk symptoms	Short–medium term	(15, 17, 31)
	Data protection and accountability	Introduce basic privacy and responsible-use principles before wider deployment	Medium term	(7, 11, 32)
	Monitoring and independent evaluation	Establish small university or multi-stakeholder groups to monitor accuracy, safety, and language performance	Medium–long term	(11, 35, 36)
	Research and local capacity building	Support locally led studies on GenAI use, cyberchondria, misinformation, and digital health inequality	Long term	(44, 46)

Quantitative indicators are presented descriptively and are drawn from the cited studies; no original statistical analysis was conducted, and estimates were not re-analyzed or statistically pooled. LLM hallucination estimates are evaluation-specific and should not be interpreted as prevalence among Somali users. Governance priorities are pragmatic, phased recommendations rather than empirically validated rankings.

## A new public-health perspective

Generative AI changes the health-information ecosystem by making health information more conversational, synthesized, and adaptive than conventional web search, shifting informational authority from lists of sources or peer networks toward AI-mediated exchanges that feel personalized and continuously responsive (4, 47). That shift creates genuine opportunity because digital health can widen access to health knowledge and reduce some information barriers, especially where formal access to care is uneven (27). It also creates new risks because generative models can fabricate or distort medical content, produce misinformation at scale, and generate fluent outputs that may appear credible despite limitations in factual or clinical reliability (4, 6).

The article’s core contribution is to argue that, in this new environment, AI-related cyberchondria should not be understood only as an individual behavioral problem. Online health searching already becomes harmful when overload, trust, and uncertainty interact to intensify anxiety and alter care-seeking patterns (37, 38). When these dynamics are mediated through more persuasive and interactive systems, they could plausibly have implications for digital public health and health-system demand, although this pathway remains partly conceptual and still requires empirical validation (1, 17). Somalia is particularly relevant here because it illustrates how technological expansion can outpace health-system capacity, local validation, and language inclusion, thereby highlighting potential links between AI-mediated information access and digital health inequality (27, 47). The appropriate response is therefore not to prevent GenAI use, but to build safe, equitable, locally validated, transparent, and accountable AI-enabled health-information ecosystems (47).

## Conclusion

Generative AI is reshaping the health-information environment by making medical information more accessible, conversational, personalized, and continuously interactive. These characteristics offer potential opportunities for resource-constrained settings, particularly through health education, translation, patient communication, and support for healthcare workers. However, generative AI systems can also produce inaccurate or misleading information, creating challenges related to information quality, uncertainty, trust, and safe decision-making.

Established research shows that repeated online health-information seeking can be associated with health anxiety, information overload, reassurance seeking, and cyberchondria. Whether these established patterns are amplified or otherwise modified by generative AI remains uncertain. The conversational, personalized, and iterative characteristics of GenAI provide a plausible mechanism through which repeated symptom searching or reassurance seeking could occur, particularly among individuals already vulnerable to health anxiety. However, direct evidence linking GenAI use to cyberchondria, and evidence linking such use to subsequent changes in healthcare utilization, remain limited.

Somalia provides an important context for investigating these questions because expanding digital connectivity intersects with unequal access to technology, constrained healthcare access, limited local health-information infrastructure, and a low-resource language environment. These structural characteristics may shape how GenAI-mediated health information is accessed and interpreted, but they should not be treated as evidence that GenAI-related cyberchondria is already occurring at the population level.

Future research should therefore test the proposed relationship empirically, including whether frequency and patterns of GenAI-mediated health-information seeking are associated with cyberchondria-related outcomes and whether these relationships vary according to health anxiety, digital literacy, socioeconomic position, language, connectivity, and access to professional healthcare. In parallel, Somali-language validation, digital and health literacy, human oversight, transparent governance, data protection, and local research capacity should be strengthened. Such an approach would allow the potential benefits of GenAI to be evaluated without prematurely assuming either harm or benefit, while ensuring that emerging AI-enabled health-information systems are safe, equitable, locally appropriate, and accountable.

## Data Availability

The original contributions presented in the study are included in the article/supplementary material, further inquiries can be directed to the corresponding author/s.
